# Repeated-Measures Implication of Hepatocellular Carcinoma Biomarkers in Living Donor Liver Transplantation

**DOI:** 10.1371/journal.pone.0124943

**Published:** 2015-05-15

**Authors:** King-Wah Chiu, Toshiaki Nakano, Kuang-Den Chen, Li-Wen Hsu, Chia-Yun Lai, Ching-Yin Huang, Yu-Fan Cheng, Shigeru Goto, Chao-Long Chen

**Affiliations:** 1 Liver Transplantation Program, Kaohsiung Chang Gung Memorial Hospital, Kaohsiung, Taiwan; 2 Chang Gung University, College of Medicine, Taoyuan, Taiwan; The University of Hong Kong, HONG KONG

## Abstract

**Objective:**

Hepatocellular carcinoma (HCC) and its recurrence are major problems in living donor liver transplantation (LDLT). Several biomarkers have been used to investigate this event. We conducted a prospective controlled study to determine the activities of the basic fibroblast growth factor (FGF-2), survivin, Ki67, endostatin, and vascular endothelial growth factor (VEGF) in different conditions before, early after, and late after LDLT with and without HCC recurrence.

**Methods:**

Fifty patients with virus-related HCC who underwent LDLT were enrolled in this 2-year cross-sectional study. During the study period, recurrent HCC was identified in 9 patients (study group, n = 9) and 41 patients (control group, n = 41) had no recurrence after LDLT. The mean time to HCC recurrence was 587.11 ± 398.64 days (range, 90–1352 days). Microvascular invasion (MVI) was found in 66.7% (6/9) of the recipients, as determined on pathological examination. The serum biomarkers were investigated by using enzyme-linked immunosorbent assay at the different LDLT stages.

**Results:**

The serum levels of the biomarkers significantly correlated with LDLT and HCC recurrence in the repeated-measures analysis (F = 31.676, P = 0.000). Significant differences were observed in the effects of all biomarkers (F = 85.313, P = 0.000) and the time to HCC recurrence after LDLT (F = 3.178, P = 0.046). The biomarkers, ordered by the observed power of the test for HCC recurrence after LDLT, were FGF-2 (1.000) > survivin (0.999) > Ki67 (0.949) > endostatin (0.411) > VEGF (0.305).

**Conclusions:**

Different biomarker activities may be implicated in the pathogenesis of HCC recurrence after LDLT. Oncogenes may not exist in the new graft but may still be present in the peripheral blood. The timing of HCC recurrence and impact of MVI in the explanted liver requires confirmation in larger studies with a longer follow-up.

## Introduction

In Taiwan, hepatocellular carcinoma (HCC) is a leading cause of cancer mortality in male patients. Living donor liver transplantation (LDLT) was reported to be potentially curative for cirrhotic patients with HCC, as it simultaneously treats both conditions [1–2]. In the liver transplantations performed at our center, abnormal liver function, acute rejection, recurrent infectious disease, or transient portal hypertension esophageal bleeding were complications observed after LDLT [3–5]. However, one of the most serious complications of LDLT is recurrent HCC, especially when Edmondson-Steiner’s grading indicates poor differentiation [6–7]. This is an issue not only for our programs but also for liver transplantation centers worldwide. In addition to alpha-fetoprotein (AFP), a number of specific biomarkers provide useful clinical information for early detection and prognosis, pathogenesis, and treatment efficacy [8]. Many tumor markers have been used as prognostic and therapeutic biomarkers. The basic fibroblast growth factor (FGF-2) is a member of the fibroblast growth factor family. It has been hypothesized that during both wound healing of normal tissues and tumor development, the action of heparan sulfate-degrading enzymes activates FGF-2, thus mediating the formation of new blood vessels in a process known as angiogenesis. Survivin is a unique member of the inhibitor of apoptosis protein family and regulates the cell cycle in most tumors. However, it is barely detectable in terminally differentiated normal cells and tissues. Differential expression of survivin in cancer compared with normal tissues makes it a useful tool in cancer diagnosis and a promising therapeutic target. The Ki-67 protein is a cellular marker for proliferation and can be used to identify the actively dividing fraction of a given cell population. The fraction of Ki-67-positive tumor cells is often correlated with the clinical course of cancer. Endostatin is a broad-spectrum angiogenesis inhibitor and may interfere with the pro-angiogenic action of growth factors such as FGF-2 and vascular endothelial growth factor (VEGF). VEGF is the most important angiogenesis factor and a highly specific mitogen for blood vessel endothelial cells. It stimulates microvessel endothelial cells to proliferate and increase permeability, resulting in tumor angiogenesis [9–13]. Based on the pathogenesis of HCC, we conducted this prospective, cross-sectional, controlled study by using FGF-2, survivin, Ki67, endostatin, and VEGF activities to determine HCC recurrence before, early after, and late after LDLT.

## Materials and Methods

### Patients

This 2-year prospective study enrolled 50 HCC-associated patients who underwent LDLT in our liver transplant programs. Before LDLT, all non-HCC-associated patients, including pediatric cases, were excluded from this study. The study group comprised nine recipients with HCC recurrence within the 2-year period after LDLT. The control group consisted of 41 recipients without HCC recurrence after LDLT. The mean age of the two groups was 58.0 and 59.0 years, respectively. The male-to-female ratios in the two groups were 9:0 and 38:3, respectively. The mean time to HCC recurrence was 587.11 ± 398.64 days (range, 90–1352 days). Microvascular invasion (MVI) to the pathological site was found in 66.7% recipients (6/9). Edmondson-Steiner’s cell grading of HCC showed that 88.9% (8/9) of patients had grade II cell differentiation. HCC recurred in 88.9% (8/9) of the cases with distant metastases, including the lung (55.6%, 5/9), adrenal gland (22.2%, 2/9), lymph nodes (22.2%, 2/9), brain (11.1%, 1/9), and bone (11.1%, 1/9). In HCC recurrence after LDLT, AFP levels remained within normal limits for 44.4% (4/9) of cases, was 2–4 times above the normal limit in 22.2% (2/9), and was more than several thousand nanograms per milliliter in 33.3% (3/9) (Table 1). At follow-up for all the study cases, the survival rate was 100% (41/41) in the control group. In contrast, 44.4% (4/9) of the patients in the study group did not survive, even with advanced treatment, and 55.6% (5/9) survived until the end of the study. Patient serum samples were analyzed by using enzyme-linked immunosorbent assay (ELISA), focusing on FGF-2, survivin, Ki67, endostatin, and VEGF at three different time periods as follows: before LDLT (preoperative day 1 as the baseline before transplantation), early after LDLT (postoperative day 1 as the baseline after liver transplantation), and late after LDLT (control group, postoperative day 30; study group, the day of the first detection of HCC recurrence during follow-up). For cases of HCC recurrence after LDLT, blood samples for before and early after LDLT were obtained from a tissue bank of our liver transplant programs within the study period and analyzed. Samples for late after LDLT were obtained at the time of documentation of HCC recurrence after LDLT. HCC patients with high AFP levels (>200 ng/mL) and poor differentiation (>grade III in Edmondson-Steiner’s cell grading) were not considered for LDLT in our program [2]. As a result, AFP was not used in our biomarker investigation. Our follow-up protocol for detection of HCC and distant metastases consisted of regular blood tests, including liver function and AFP analysis every month, ultrasonography and chest radiography every 3 months, and computed tomography of the liver and chest every year. In additional to the chest and brain magnetic resonance imaging, computed tomography was performed when the AFP level was elevated or ultrasonography and/or chest radiography results showed abnormal findings.

**Table 1:** Clinical data of the nine living donor liver transplant recipients with recurrence of hepatocellular carcinoma.

No.	Outcome	Time to Recurrence (Days)*	Sites of Recurrence	Serum AFP Level (ng/dL) in LDLT			MVI	Cell Grade^#^
				Before	Early After	Documented Recurrence		
1	expired	793	lung/adrenal/retrocaval	<20	<20	2186	-	II
2	survival	1352	liver/adrenal	<20	<20	212751	+	II
3	expired	669	lung/brain	<20	<20	71	+	II
4	survival	270	liver, multiple	<20	<20	<20	-	II
5	expired	365	bone/spine	<20	<20	<20	+	II
6	survival	255	lung	<20	<20	<20	-	I
7	survival	945	liver	<20	<20	44	+	II
8	survival	545	lung	<20	<20	<20	+	II
9	expired	90	lung/lymph nodes	<20	<20	81822	+	II
Mean ± 2 SD		587.11 ± 398.64 days						

*Days from LDLT to HCC recurrence.

^#^Edmondson-Steiner’s cell grading of HCC. AFP, alpha-fetoprotein; HCC, hepatocellular carcinoma; LDLT, living donor liver transplantation; MVI, microvascular invasion in the original removed liver, 66.7% (6/9); SD, standard deviation

### Fibroblast growth factor 2

Serum FGF-2 levels were measured by using an ELISA measurement kit (R&D Systems, Minneapolis, MN, USA). Each well containing a precipitate was mixed well with 100 μL of the assay diluent HD1-46. Next, 150 μL of standard, control, or sample specimen was added to each well. Wells were covered with the provided adhesive strip and incubated for 3 h at room temperature (18–23°C). A plate layout was provided as a record of the standards, and the samples were assayed. FGF basic conjugate (200 μL) was added to each well, the plate was covered with a new adhesive strip, and incubated for 2 h at room temperature. Substrate (50 μL) was added to each well, covered with a new adhesive strip, and incubated for 45 min at room temperature. Amplifier solution (50 μL) was added to each well, and the plate was covered with a new adhesive strip. Finally, 50 μL of stop solution was added to each well. The optical density of each well was determined within 30 min. Absorbance at 450 nm of the colored reaction product was analyzed with an ELISA microplate reader. All serum samples were assayed in duplicate by one operator in order to assess interassay precision. Results were calculated from a standard curve generated by a parametric logistic curve fit and expressed in picogram per milliliter serum.

### Survivin

Serum survivin was measured manually by using ELISA with Quantikine kits (R&D Systems). Standard dilutent (100 μL) was pipetted into the appropriate wells, and the plate was gently tapped to mix the contents. The plate was sealed and incubated at room temperature on a plate shaker for 1 h at 500 rpm. Next, 100 μL of antibody was pipetted into each well, and the plate was sealed and incubated at room temperature on a plate shaker for 1 h at 500 rpm. Conjugate (100 μL) was added to each well, and the plate was sealed and incubated at room temperature on a plate shaker for 30 min at 500 rpm. Substrate (100 μL) was pipetted into each well and incubated for 30 min at room temperature on a plate shaker at 500 rpm. Finally, 100 μL of stop solution 2 was pipetted into each well. The optical density at 450 nm was analyzed by using a LabSystems Multiskan Spectrum Microplate Reader (Thermo Scientific, Waltham, MA, USA), according to the manufacturer’s protocol. Results were calculated from a standard curve generated by a parametric logistic curve fit and expressed in pictogram per milliliter serum.

### Ki67

Serum Ki67 was measured by using a commercially available Human Antigen Ki-67 ELISA kit (Cusabio Biotech Co., LTD, Wuhan, Hubei, China) based on a sandwich ELISA. Results were calculated from a standard curve generated by a parametric logistic curve fit and expressed in nanograms per milliliter serum. Standard (100 μL) and sample specimens were added to each well and incubated for 2 h at 37°C. Next, 100 μL of prepared detection reagent A was added and incubated for 1 h at 37°C. Detection reagent B (100 μL) was added and incubated for 30 min at 37°C. Substrate (90 μL) was added and incubated for 15–25 min at 37°C. Finally, 50 μL of stop solution was added. Absorbance at 450 nm was immediately analyzed in a microplate reader.

### Endostatin

Serum endostatin was measured by using a commercially available ELISA kit for endostatin (R&D Systems). Results were calculated from a standard curve generated by a parametric logistic curve fit and expressed in nanograms per milliliter serum. Samples were mixed with 100 μL of assay diluent RD1W, covered with the provided adhesive strip and incubated for 2 h at room temperature on a horizontal orbital microplate shaker set at 500 rpm. The standards were recorded on a plate layout, and the samples were assayed. Next, conjugated endostatin (200 μL) was added to each well, and the plate was covered with a new adhesive strip and incubated for 2 h at room temperature on a shaker. Substrate (200 μL) was added to each well and incubated in the dark for 30 min at room temperature. The reaction was stopped with 50 μL of stop solution, and the plate was tapped to ensure thorough mixing. The optical density at 450 nm of each well was determined within 30 min by using a microplate reader.

### Vascular endothelial growth factor

VEGF level was measured by using a commercial ELISA kit for VEGF (R&D Systems), following the manufacturer’s instructions. Results from a standard curve generated by a parametric curve fit were calculated and expressed in pictogram per milliliter serum. An ELISA plate was coated with 100-μL/well capture antibody overnight at room temperature. Each well was washed with wash buffer (400 μL) by using a squirt bottle and an electrical single channel pipette. Plates were blocked by adding to each well 300 μL of phosphate-buffered saline containing 1% bovine serum albumin, 5% sucrose, and 0.05% NaN3 and incubated at room temperature for at least 1 h. The samples and standards were diluted in polypropylene tubes, and 100 μL of sample or standard was added to each well. The plate was mixed by gentle tapping for 1 min, covered with an adhesive strip, and incubated for 2 h at room temperature. A biotinylated detection antibody was diluted, and 100 μL was added to each well. The plate was covered with a new adhesive strip and incubated for 2 h at room temperature. Streptavidin conjugated with horseradish peroxidase (100 μL) was added to each well, and the plate was covered and incubated for 20 min at room temperature. Substrate (100 μL) was added to each well and incubated in the dark for 20–30 min at room temperature. Finally, 50 μL of stop solution was added to each well, and the plate was gently tapped to ensure thorough mixing. The optical density of each well at 450 nm was determined within 30 min by using a microplate reader.

For each assay, baseline and post-intervention samples from the same individual were analyzed in the same experiment. Each sample was assayed in duplicate by one operator to assess interassay precision.

### Ethics statement

All of the participants provided written informed consent to participate in this study. The ethics committees approved all consent procedures. Ethical approval was obtained for association and institutional standards by the institute review board of Chang Gung Memorial Hospital. The ethics committee specifically approved the complete study (No. 103-0352C). None of the transplant donors were from a vulnerable population, and all of the donors or their next of kin freely provided written informed consent.

### Statistical analysis

Data were analyzed by using the Statistical Package for repeated measures (SPSS v. 22.0 for Windows, IBM Corp., Armonk, NY, USA). Results were obtained from comparisons of between- and within-subject effects, partial *η* ^2^, and observed power. The Mann-Whitney *U* test was used to compare the difference in biomarkers between the patients with and those without HCC recurrence. Differences were considered statistically significant when *P* < 0.05.

## Results

**Fig 1:**
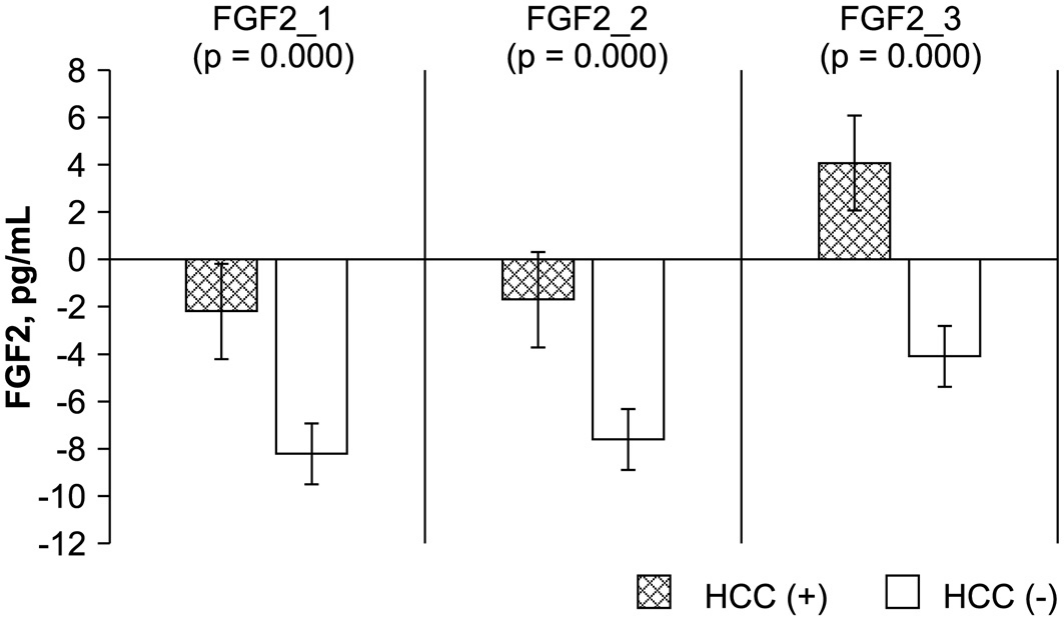
Statistical analysis of FGF-2, survivin, Ki67, endostatin, and VEGF levels, as measured by ELISA before (_1), early after (_2), and late after (_3) after living donor liver transplantation (LDLT). The Mann-Whitney *U* test was used to compare the differences in the biomarkers at the various time points between the recipients with and those without HCC recurrence.

**Fig 2:**
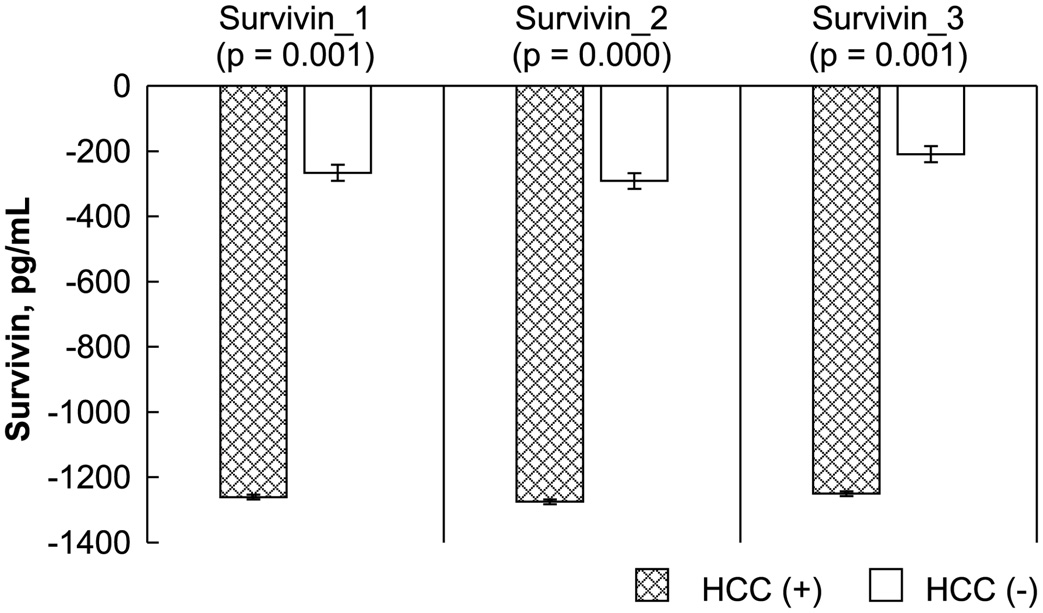
Statistical analysis of FGF-2, survivin, Ki67, endostatin, and VEGF levels, as measured by ELISA before (_1), early after (_2), and late after (_3) after living donor liver transplantation (LDLT). The Mann-Whitney *U* test was used to compare the differences in the biomarkers at the various time points between the recipients with and those without HCC recurrence.

**Fig 3:**
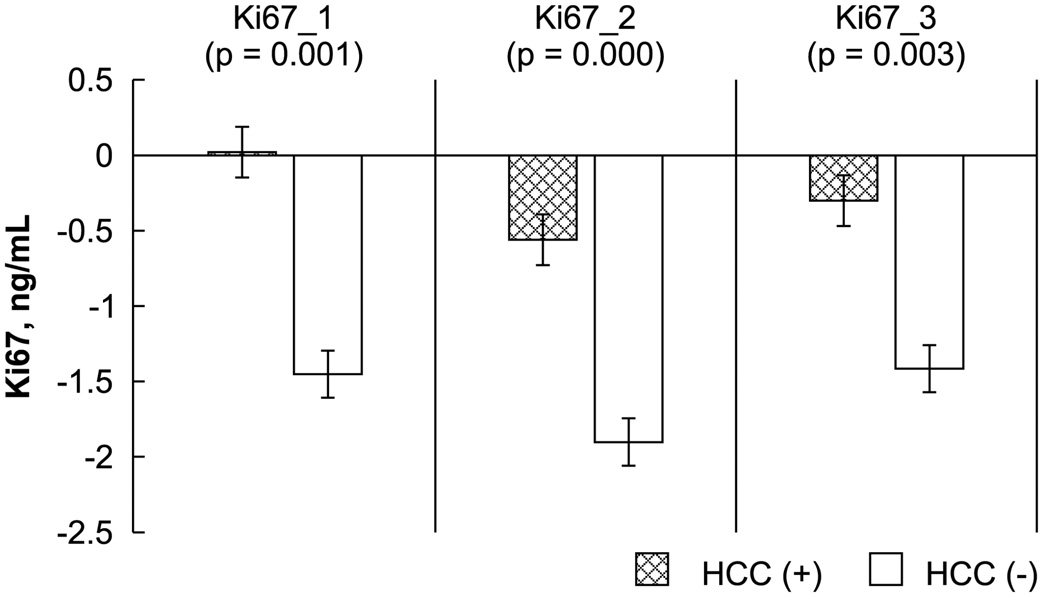
Statistical analysis of FGF-2, survivin, Ki67, endostatin, and VEGF levels, as measured by ELISA before (_1), early after (_2), and late after (_3) after living donor liver transplantation (LDLT). The Mann-Whitney *U* test was used to compare the differences in the biomarkers at the various time points between the recipients with and those without HCC recurrence.

**Fig 4:**
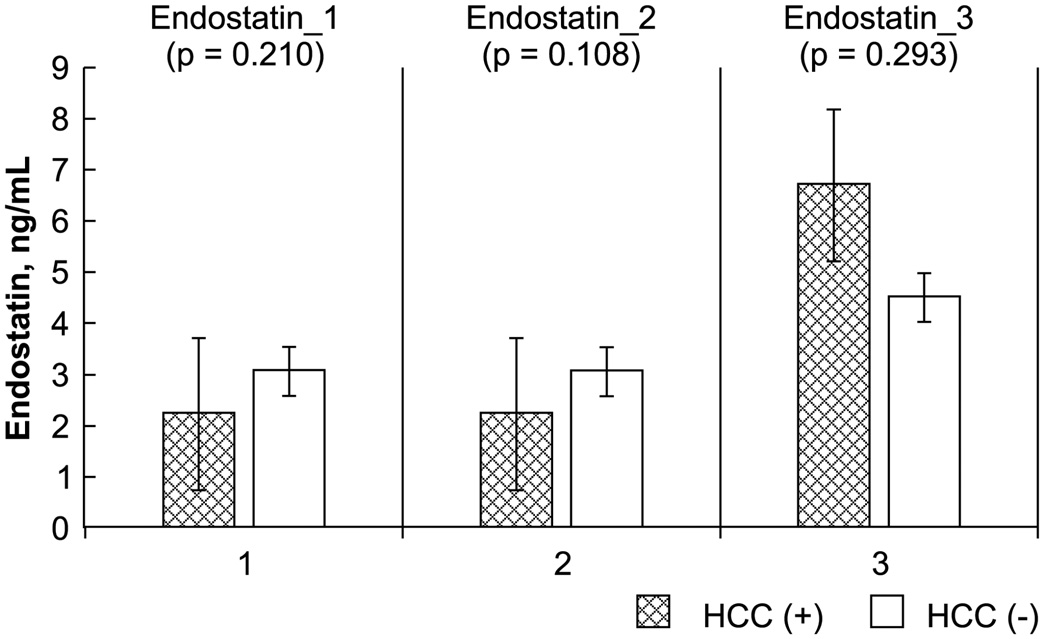
Statistical analysis of FGF-2, survivin, Ki67, endostatin, and VEGF levels, as measured by ELISA before (_1), early after (_2), and late after (_3) after living donor liver transplantation (LDLT). The Mann-Whitney *U* test was used to compare the differences in the biomarkers at the various time points between the recipients with and those without HCC recurrence.

**Fig 5:**
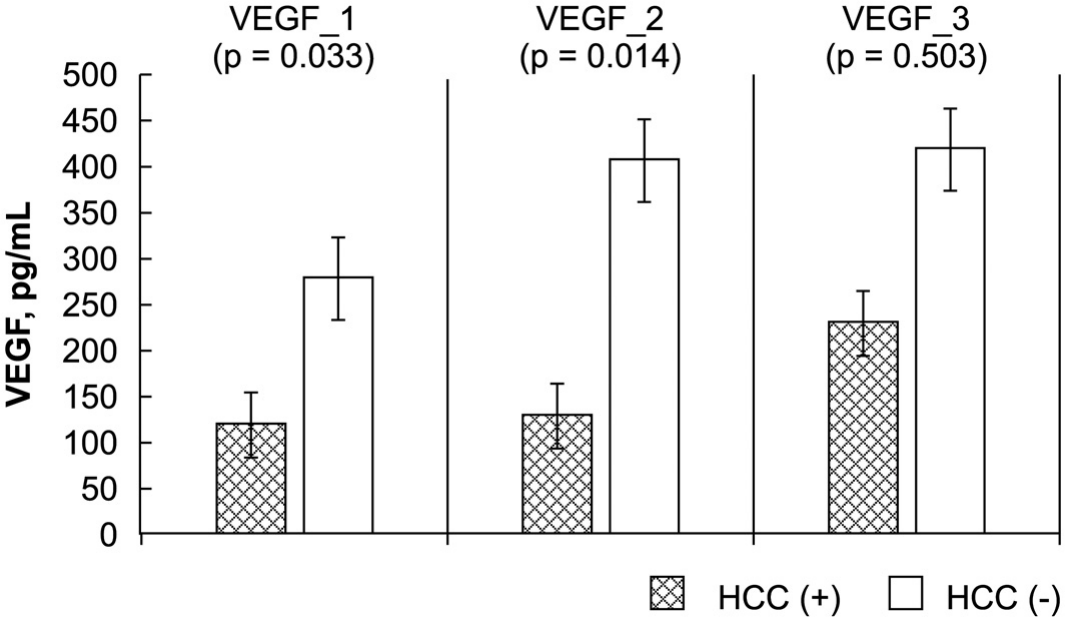
Statistical analysis of FGF-2, survivin, Ki67, endostatin, and VEGF levels, as measured by ELISA before (_1), early after (_2), and late after (_3) after living donor liver transplantation (LDLT). The Mann-Whitney *U* test was used to compare the differences in the biomarkers at the various time points between the recipients with and those without HCC recurrence.

The levels of the HCC biomarkers FGF-2, survivin, Ki67, endostatin, and VEGF between the study and control groups before, early after, and late after LDLT were analyzed by ELISA. The Mann-Whitney *U* test results showed a significant difference between the study (recurrent HCC) and control (no recurrence) groups before, early after, and late after LDLT for FGF-2 (-4.14 ± 2.38 vs. -11.25 ± 4.98 pg/mL, P = 0.000; -4.45 ± 3.68 vs. -10.20 ± 4.19 pg/mL, P = 0.000; 0.91 ± 3.85 vs. -8.22 ± 4.76, P = 0.000, respectively), survivin (-1261.09 ± 68.89 vs. -266.44 ± 519.86 pg/mL, P = 0.000; -1275.07 ± 59.25 vs. -291.39 ± 476.80 pg/mL, P = 0.000; -1250.34 ± 70.57 vs. -209.19 ± 593.00 pg/mL, P = 0.000, respectively), and Ki67 (0.02 ± 0.68 vs. -1.45 ± 1.02 ng/mL, P = 0.001; -0.56 ± 0.40 vs. -1.90 ± 0.76 ng/mL, P = 0.000; -0.30 ± 0.56 vs. -1.41 ± 0.87 ng/mL, P = 0.003, respectively). Differences in VEGF before and early after LDLT were also significant (119.29 ± 53.10 vs. 278.37 ± 195.49 ng/mL, P = 0.033; 128.92 ± 71.85 vs. 406.45 ± 338.43 ng/mL, P = 0.014). However, no significant differences were observed between the patients with and those without HCC recurrence in terms of endostatin level before, early after, or late after LDLT (2.23 ± 2.68 vs. 3.07 ± 1.45 ng/mL, P = 0.210; 2.23 ± 2.35 vs. 3.06 ± 1.47 ng/mL, P = 0.108; 6.69 ± 4.76 vs. 4.50 ± 3.01 ng/mL, P = 0.293, respectively) or in VEGF late after LDLT (229.70 ± 85.28 vs. 418.52 ± 378.24 pg/mL, P = 0.503) (Figs 1–5).

The observed power of the repeated-measures analysis of the biomarker levels late after LDLT with HCC recurrence showed that the power of FGF-2 (1.000) was > survivin (0.999) > Ki67 (0.949) > endostatin (0.411) > VEGF (0.305) (Table 2). Tests of the between-subjects effects on HCC recurrence revealed that all biomarkers were significant (F = 31.676, P = 0.000, partial *η* ^2^ = 0.398, observed power = 1.000) (Table 3). The results of the tests of within-subjects effects showed a strong correlation to the biomarkers (F = 85.313, P = 0.000, partial *η* ^2^ = 0.640, observed power = 1.000), but a weak correlation to time (F = 3.178, P = 0.046, partial *η* ^2^ = 0.062, observed power = 0.596) (Table 4).

**Table 2:** Observed power of hepatocellular carcinoma biomarkers in living donor liver transplantation.

Dependent variable	HCC	B	Standard Error	*t*	*P*	95% Confidence Interval		Partial *η* ^2^	Observed Power^a^
						Lower Bound	Upper Bound		
FGF-2_1	-	-4.137	1.549	-2.670	.010	-7.252	-1.022	0.129	0.744
	+	-7.115	1.711	-4.159	.000	-10.555	-3.675	0.265	0.983
FGF-2_2	-	-4.450	1.370	-3.249	.002	-7.204	-1.696	0.180	0.889
	+	-5.749	1.513	-3.801	.000	-8.790	-2.708	0.231	0.961
FGF-2_3	-	.910	1.541	.591	.557	-2.187	4.008	0.007	0.089
	+	-9.127	1.701	-5.365	.000	-12.547	-5.706	0.375	**1.000**
Survivin_1	-	-1261.093	158.464	-7.958	.000	-1579.707	-942.479	0.569	1.000
	+	994.654	174.995	5.684	.000	642.804	1346.504	0.402	1.000
Survivin_2	-	-1275.066	145.310	-8.775	.000	-1567.231	-982.902	0.616	1.000
	+	983.678	160.468	6.130	.000	661.035	1306.320	0.439	1.000
Survivin_3	-	-1250.338	180.701	-6.919	.000	-1613.662	-887.015	0.499	1.000
	+	1041.144	199.551	5.217	.000	639.921	1442.367	0.362	**0.999**
Ki67_1	-	.021	.323	.064	.949	-.628	.670	0.000	0.050
	+	-1.471	.356	-4.127	.000	-2.188	-.754	0.262	0.981
Ki67_2	-	-.560	.237	-2.363	.022	-1.036	-.084	0.104	0.639
	+	-1.342	.262	-5.128	.000	-1.868	-.816	0.354	0.999
Ki67_3	-	-.301	.275	-1.095	.279	-.853	.251	0.024	0.189
	+	-1.114	.303	-3.672	.001	-1.724	-.504	0.219	**0.949**
Endostatin_1	-	2.228	.573	3.887	.000	1.076	3.381	0.239	0.968
	+	.839	.633	1.325	.191	-.434	2.112	0.035	0.255
Endostatin_2	-	2.230	.550	4.056	.000	1.125	3.335	0.255	0.978
	+	.828	.607	1.364	.179	-.392	2.049	0.037	0.267
Endostatin_3	-	6.693	1.123	5.962	.000	4.436	8.950	0.425	1.000
	+	-2.195	1.240	-1.771	.083	-4.688	.297	0.061	**0.411**
VEGF_1	-	119.293	59.922	1.991	.052	-1.188	239.774	0.076	0.496
	+	159.078	66.173	2.404	.020	26.029	292.127	0.107	0.654
VEGF_2	-	128.922	103.443	1.246	.219	-79.064	336.907	0.031	0.231
	+	277.529	114.233	2.429	.019	47.847	507.210	0.110	0.663
VEGF_3	-	229.701	115.677	1.986	.053	-2.884	462.285	0.076	0.494
	+	188.820	127.744	1.478	.146	-68.027	445.667	0.044	**0.305**

^a^Computed using = 0.05; _1, before LDLT. _2, early after LDLT; _3, later after LDLT; -, without HCC recurrence; +, with HCC recurrence. FGF-2, basic fibroblast growth factor; HCC, hepatocellular carcinoma; LDLT, living donor liver transplantation; Std. Error, standard error; VEGF, vascular endothelial growth factor

**Table 3:** Tests of between-subjects effects on biomarkers in living donor liver transplantation with hepatocellular carcinoma recurrence.

Biomarkers	Source	Type III Sum of Squares	*df*	Mean Square	F	*P*	Partial *η* ^2^	Observed Power
FGF-2, endostatin and VEGF	Intercept	2010125.480	1	2010125.480	37.939	0.000	0.441	1.000
	HCC	298067.931	1	298067.931	5.626	0.022	0.105	0.642
	Error	2543208.844	48	52983.518				
Survivin and Ki67	Intercept	25566333.310	1	25566333.310	77.347	0.000	0.617	1.000
	HCC	11185048.287	1	11185048.287	33.838	0.000	0.413	1.000
	Error	15866037.997	48	330542.458				
FGF-2, survivin, Ki67, endostatin, and VEGF	Intercept	4408658.406	1	4408658.406	21.678	0.000	0.311	0.995
	HCC	6441867.629	1	6441867.629	31.676	0.000	0.398	1.000
	Error	9761759.309	48	203369.986				

FGF-2, basic fibroblast growth factor; VEGF, vascular endothelial growth factor.

**Table 4:** Results of the tests of within-subjects effects on biomarkers and time in living donor liver transplantation with hepatocellular carcinoma recurrence.

Source	Type III Sum of Squares	*df*	Mean Square	F	*P*	Partial *η* ^2^	Observed Power^(^ ^a^ ^)^
Biomarkers	52753920.376	4	13188480.094	85.313	0.000	0.640	1.000
Biomarkers * HCC	16949998.585	4	4237499.646	27.411	0.000	0.363	1.000
Time-period	85968.604	2	42984.302	3.178	0.046	0.062	0.596
Time-period * HCC	9048.117	2	4524.059	.334	0.717	0.007	0.102
Biomarkers * Time-period	190140.417	8	23767.552	1.725	0.091	0.035	0.748
Biomarkers * Time-period * HCC	60826.552	8	7603.319	.552	0.817	0.011	0.257

^a^: computed using alpha = 0.05

## Discussion

Based on the pathogenesis of HCC, the identification of significant factors and the mechanisms of HCC recurrence after LDLT remain major challenges in contemporary biomedicine. In the period between LDLT and HCC recurrence, timing after transplantation was found to be a potential influencing factor and is one of the factors investigated here. In our study, the mean time to HCC recurrence was 587.11 ± 398.64 days. Liver grafts were obtained from donors without HCC, and the circulating individual oncogene could be identified from peripheral blood but not from the liver graft at the time of HCC recurrence. We used five biomarkers reported to be associated to HCC to investigate HCC recurrence at different times after LDLT. These biomarkers were the growth factor FGF-2, the angiogenesis factors VEGF and endostatin, and the apoptosis- and cancer cell proliferation-related proteins survivin and Ki67. A significant difference was observed between the groups with and without HCC recurrence in terms of serum FGF-2, survivin, and Ki67 levels at different time points (P < 0.000). This is further evidence that HCC recurrence at different time points is associated with the activity of different biomarkers in circulating blood [14]. In our recent study, in donor grafts, specific protein expression did not change in the peripheral blood in the LDLT recipients [15] and was homogenous in the new liver [16–18]. One possible reason is that most HCC recurrences in the present study were isolated distant metastases (77.8%). Although VEGF has been documented in HCC development and prognosis [13], VEGF activity did not obscure differences in the comparison between the study and control groups in an individual paired *t* test analysis. For both HCC recurrence and liver graft regeneration after LDLT, angiogenesis and the VEGF receptor pathways might be activated, possibly resulting in the loosening of intercellular junctions of endothelial cells and promoting angiogenesis. The VEGF receptor pathway plays a physiological role that may not differentiate between HCC recurrence and liver graft regeneration after LDLT [19–20]. In our experience, liver graft regeneration requires significant blood flow [21] and leads to the activation of this pathway. Studies performed under the same conditions suggest that serum endostatin is also significantly related to liver regeneration in an animal model [22]. To determine the effect of biomarkers on HCC recurrence over time, a repeated-measures analysis of variance has been recommended as the best method of investigation [23]. Analysis of variance is a generalized method of the paired *t* test. The only difference between these methods is that, for the former, measurements were taken in the same individuals. The measurements are likely to be correlated, and any analysis must consider such correlations. The advantages of this statistical method are that subjects are also the controls themselves, isolating variability between subjects, and the analysis can focus more precisely on recurrent effects. As the present study is a 2-year prospective study with a limited number of cases of HCC recurrence after LDLT, the repeated-measures design is suitable. Because the subjects were also the controls themselves, fewer subjects are needed [24]. A repeated-measures analysis of variance (ANOVA) may be used when the same parameter has been measured under different conditions in the same subjects. Subjects can be divided into different groups, as in the present study. Performing a repeated-measures analysis can overcome the statistical limitations in cases with HCC recurrence over time and reduce bias from individual biomarker analyses. In the tests of between-subjects effects, all of the biomarkers reached a level of significance (0.000) and were involved in HCC recurrences after LDLT, with an observed power of up to 1.000 (Table 3). We demonstrated how invariance between and within subjects can be tested by high-frequency repeated measures in this study. Because individual biomarker activities may be expressed differently after LDLT in individual recipients over time, a simple paired *t* test would be a biased description of this phenomenon. Meanwhile, the results of the tests of within-subjects effects in our study showed that the biomarkers were significantly involved in HCC recurrence after LDLT and further showed that time was one of the influencing factors (Table 4). This approach also solved the bias problem in studies with a small number of cases. When HCC recurs late after LDLT, this can be determined from the observed biomarker levels that reflect the bioactivity of the liver [21–22]. Measures of effect size in repeated-measures ANOVA indicate the degree of association between an effect and its dependent variable. Partial *η* ^2^ is a common measure of effect size and estimates of the degree of association of the sample. In Table 2, partial *η* ^2^ was 0.375 in FGF-2_3, representing an effect size of 37.5% in the relationship between the value and HCC recurrence. The observed power was 1.000, much higher than that of other biomarkers. In this study, the bioactivity of FGF-2 was > survivin > Ki67 > endostatin > VEGF during HCC recurrence after LDLT (Table 2). Although we found that MVI does not influence patient outcome after LDLT [1], 77.8% of patients with HCC recurrence presented with distant metastasis, of which 66.7% were associated with MVI in the explanted liver (Table 1). Given that circulation biomarkers have been previously identified from peripheral blood, it is likely that MVI influences recurrence after LDLT [25–26]. A recent study showed that higher VEGF plasma levels before liver transplantation may influence HCC recurrence [27]. In our study, we found a significant difference (P = 0.033) in serum VEGF level by using the Mann-Whitney *U* test of the data of the samples obtained before LDLT. After liver transplantation, immunosuppressive agents such as sirolimus downregulate mRNA expression of VEGF, but not that of FGF-2 [28]. This may be one of the reasons for the lack of significant differences in VEGF between samples collected early and late after LDLT. In contrast, FGF-2 level was found to have a higher observed power in the tests of between-subjects effects in all the studied biomarkers. Survivin is critical in promoting cell proliferation [29], and all cell lines stain strongly for Ki67 [30]. These three biomarkers could be important biomarkers for detection of HCC recurrence after LDLT.

In conclusion, the present study indicates that HCC recurrence is defined by the activity of biomarkers such as FGF-2, survivin, Ki67, endostatin, and VEGF. This activity is naturally differs between individuals and becomes more complex over time. It is difficult to predict HCC recurrence with a single variable. However, a repeated-measures analysis shows the fluctuation of multiple biomarkers over time after LDLR and may predict HCC recurrence and pathogenesis. Oncogenes are not present in the new graft but appear to remain in peripheral blood. The timing of HCC recurrence and the impact of MVI in the explanted liver requires confirmation in larger studies with longer follow-up periods.
